# Supplementary material for: Utilizing protein structure to identify non-random somatic mutations
Source: BMC Bioinformatics. 2013 Jun 13;14:190. doi: 10.1186/1471-2105-14-190 (PMC3691676; doi:10.1186/1471-2105-14-190)
Supplement: Additional file 1 — Cosmic Query. The SQL query used to extract the mutations from COSMIC. [file 1471-2105-14-190-S1.docx]

Below is the SQL query used to generate the raw data from Cosmic Database version 58.

drop table test;

create table test

as

SELECT GSO.gene_name,SA.id_sample, upper(REPLACE(sa.sample_name, '-','')) as sample_name,gsm.id_mutation, SM.AA_mut_start, SM.AA_MUT_STOP,length(TR. TRANSCRIPT_AA_SEQ) as AA_len, GSO.swissprot_accession,

SA.ID_SOURCE_SPEC,SA.ID_source, GSM.ID_MUT_SOMATIC_STATUS, gsm.ID_MUT_VERIF_STATUS, SA.ID_SOURCE_TISSUE_ORIGIN

FROM ANALYSED_GENE_SAMPLE T, GENE_STUDY GS, Gene_sample_mutation GSM, sequence_mutation SM ,cosmic_tumour_sample_overview SA, gene_study_transcript GST, transcript TR, Gene_SOM GSO

where GS.id_gene_study = T.id_gene_study and GSM.id_ags=T.ID_AGS and SM.id_mutation=GSM.id_mutation and SA.id_sample=T.id_sample and GST.id_gene_study=GS.id_gene_study and TR.id_transcript = GST.ID_TRANSCRIPT and GSO.id_gene=TR.id_gene

and GS.WHOLE_GENE_SCREEN='y' and GSM.ID_MUT_SOMATIC_STATUS in (1, 2, 5) and SM.id_mut_type_AA=22

order by gs.id_gene,SM.AA_mut_start,sa.sample_name;

delete from test

where (id_sample,id_mutation,aa_mut_start) in (select id_sample,id_mutation,aa_mut_start

from test group by id_sample,id_mutation,aa_mut_start having count(*)>1) and rowid not in (select min(rowid) from test group by id_sample,id_mutation,aa_mut_start having count(*)>1);

select * from test;

A visual representation of the table relationships within the database:

ID_TRANSCRIPT

ID_GENE

TRANSCRIPT_AA_SEQ

....

TRANSCRIPT

ID_GENE

GENE_NAME

CHROMOSOME

GENE_SOM

ID_GENE_STUDY

ID_GENE

ID_GENE_FUSION

ID_GENE_STUDY_TYPE

ID_PAPER

GENE_STUDY

ID_AGS

ID_GENE_STUDY

ID_SAMPLE

LOH

PAPER_SAMPLE_NUMBER

ANALYSED_GENE_SAMPLE

ID_AGS

ID_MUTATION

PERCENT_MUT_ALLELE

ID_MUT_SOMATIC_STATUS

GENE_SAMPLE_MUTATION

ID_SAMPLE

ID_TUMOUR

ID_SOURCE

IS_CGP

ID_IND....

COSMIC_TUMOUR_SAMPLE_OVERVIEW

ID_GENE_STUDY

ID_TRANSCRIPT

GENE_STUDY_TRANSCRIPT

ID_MUTATION

ID_TRANSCRIPT

ID_MUT_TYPE

ID_MUT_TYPE_AA

...

SEQUENCE_MUTATION

Restrictions imposed upon the mutation accepted for the study.

1) COSMIC studies that come to sequencing the whole gene: gene_study.whole_gene_screen = 'y')

2) Variants that are somatic: GSM.ID_MUT_SOMATIC_STATUS in (1, 2). Dictionary of mutation status is shown in table 1.

Table 1. Mutation status dictionary in mut_status_dic.

| 1 | Confirmed somatic variant | somatic_status |
| --- | --- | --- |
| 2 | Reported in another cancer sample as somatic | somatic_status |
| 3 | Confirmed germline variant | somatic_status |
| 21 | Likely cancer causing | consequence |
| 22 | Possible cancer causing | consequence |
| 23 | Unknown consequence | consequence |
| 0 | Not specified | somatic_status |
| 4 | Reported in another sample as germline | somatic_status |
| 5 | Variant of unknown origin | somatic_status |
| 6 | Not curated | somatic_status |
| 25 | To be decided | consequence |

3) Mutation type:

Substitution – missense: SM.id_mut_type_AA=22

Table 2. Part of mutation type dictionary in mut_type_dic

| 11 | Insertion |
| --- | --- |
| 12 | Deletion |
| 13 | Complex |
| 32 | Nonstop extension |
| 21 | Substitution - Nonsense |
| 22 | Substitution - Missense |
| 23 | Substitution - coding silent |
| 24 | Insertion - In frame |
| 25 | Insertion - Frameshift |

4) Delete the duplicated rows that have the same gene name, sample number and mutation positions.
